# Assessment of Antioxidant Properties in Fruits of *Myrica esculenta*: A Popular Wild Edible Species in Indian Himalayan Region

**DOI:** 10.1093/ecam/neq055

**Published:** 2011-03-17

**Authors:** Sandeep Rawat, Arun Jugran, Lalit Giri, Indra D. Bhatt, Ranbeer S. Rawal

**Affiliations:** G. B. Pant Institute of Himalayan Environment and Development, Kosi-Katarmal, Almora-263 643, Uttarakhand, India

## Abstract

Crude extract of *Myrica esculenta* fruits, a wild edible species of Indian Himalayan Region, was evaluated for phenolic compounds and antioxidant properties. Results revealed significant variation in total phenolic and flavonoid contents across populations. Among populations, total phenolic content varied between 1.78 and 2.51 mg gallic acid equivalent/g fresh weight (fw) of fruits and total flavonoids ranged between 1.31 and 1.59 mg quercetin equivalent/g fw. Antioxidant activity determined by 2,2′-azinobis(3-ethylbenzothiazoline-6-sulphonic acid) radical scavenging, 1,1-diphenyl-2-picrylhydrazyl radical scavenging and ferric reducing antioxidant power (FRAP) exhibited considerable antioxidant potential and showed significant positive correlation with total phenolic and total flavonoids content. High performance liquid chromatography analysis revealed significant variation (*P* <  .01) in phenolic compounds (i.e., gallic acid, catechin, hydroxybenzioc acid and **ρ**-coumaric acid) across populations. This study provides evidences to establish that consumption of *M. esculenta* fruits while providing relished taste would also help in reduction of free radicals. Therefore, this wild edible species deserves promotion in the region through horticulture and forestry interventions.

## 1. Introduction

Consumption of fruits and vegetables is known to lower risk of several oxidative stresses, including cardiovascular diseases, cancer and stroke [1] and such health benefits are mainly ascribed to phytochemicals such as polyphenols, carotenoids and vitamin C [2]. Of these phytochemicals, polyphenols are largely recognized as anti-inflammatory, antiviral, antimicrobial and antioxidant agents [3].

Considering above facts, besides the traditional commercial fruits, the wild fruits are also gaining increased attention as potential food supplement or cheaper alternative of commercial fruits across the world. Evidences of the health benefits of wild edible fruits, in addition to established role in nutrition are available [4]. In general, plethora of information is available on the antioxidant potential of fruits of different species. For example, *Actinidia eriantha, A. deliciosa* [5], *Ficus carica* [6], *Ficus microcarpa* [7], *Ficus racemosa* [8], *Juglans regia* [9], *Kadsura coccinea* [10], *Litchi chinensis* [11], *Morus alba* [12], *Myrciaria dubia* [13], *Nocciola piemonte* [14], *Phyllanthus emblica* [15], *Punica granatum* [16], *Randia echinocarpa* [17], *Ziziphus mauritiana* [18] and so forth. Beside the fruits, antioxidant properties are also known for other plant parts [19, 20].

In the Indian Himalayan Region (IHR) over 675 wild edibles are known [21] of which *Myrica esculenta* Buch.–Ham. ex D. Don (family Myricaceae), commonly known as “Kaphal", is amongst highly valued wild edible fruits growing between 900 and 2100 m above sea level (asl). Species is distributed from Ravi eastward to Assam, Khasi, Jaintia, Naga and Lushi hills and extends to Malaya, Singapore, China and Japan [22]. It is popular among local inhabitants for its delicious fruits and processed products [23]. This species broadly resembles with *Myrica rubra*, found commonly in China and Japan. However, *M. esculenta* contains smaller fruits of around 4-5 mm as compared with 12–15 mm fruits *of M. rubra* [24]. While information is available on phenolic contents, flavonoids, anthocyanins and antioxidant activity of *M. rubra* fruit extract, juice, jam and pomace [25–29], such information is lacking for *M. esculenta*. This study, therefore, targets *M. esculenta* fruits for assessment of total phenolics, flavonoids and phenolic compounds; evaluate range of variation in antioxidant activity using different *in vitro* methods and identify the best fruit provenance.

## 2. Methods

### 2.1. Plant Material

The ripened fruits of *M. esculenta* were collected during May–June 2008, from distantly located wild populations (i.e., Kalika (1775), Ayarpani (1950), Panuwanaula (1800), Jalna (1925), Dholichina (1950), Khirshu (1650), Shyamkhet (1975), Gwaldom (1925) and Doonagiri (2100 m asl)) in Uttarakhand, India. Immediately after collection, fruits were brought to the laboratory and kept in freezer at –4°C. The voucher specimens of the species were deposited in the herbarium of G. B. Pant Institute of Himalayan Environment and Development, Kosi-Katarmal, Almora.

### 2.2. Chemicals and Reagents

1,1-Diphenyl-2-picrylhydrazyl (DPPH) radical, gallic acid, ascorbic acid, chlorogenic acid, caffeic acid, *ρ*-coumaric acid, 3-hydroxybenzoic acid, catechin and quercetin were procured from Sigma-Aldrich (Steinheim, Germany). Sodium carbonate, 2-(*n*-morpholino) ethanesulfonic acid (MES buffer), potassium persulphate, ferric chloride, sodium acetate, potassium acetate, aluminium chloride, glacial acetic acid and hydrochloric acid from Qualigens (Mumbai, India), and 2,2′-azinobis(3-ethylbenzothiazoline-6-sulphonic acid) (ABTS), 2,4,6-tri-2-pyridyl-1,3,5-triazin (TPTZ), methanol and ethanol from Merck Company (Darmstadt, Germany).

### 2.3. Extract Preparation for Total Phenolics, Flavonoids and Antioxidant Properties

Fresh fruits (20 g) from each population were used for preparation of extract. Pulp of the fruits was carefully removed from seed and kept for continuous stirring with 50 mL (80% v/v) methanol for 24 h. Extract was filtered and filtrate was centrifuged at 8000 rpm for 10 min. Supernatant was stored at 4°C prior to use within 2 days.

### 2.4. Determination of Total Phenolics

Total phenolic content in the methanolic extract was determined by Folin-Ciocalteu's calorimetric method [30]. In 0.25 mL of diluted methanolic extract, 2.25 mL distilled water and 0.25 mL Folin-Ciocalteu's reagent was added and allowed to stand for reaction upto 5 min. This mixture was neutralized by 2.50 mL of 7% sodium carbonate (w/v) and kept in dark at room temperature for 90 min. The absorbance of resulting blue color was measured at 765 nm using UV-VIS spectrophotometer (Hitachi U-2001). Quantification was done on the basis of standard curve of gallic acid prepared in 80% methanol (v/v) and results were expressed in milligrams gallic acid equivalent (GAE) per gram fresh weight (fw) of fruits.

### 2.5. Determination of Total Flavonoids

Flavonoid content in the methanolic extract of plant was determined by aluminium chloride calorimetric method [31]. Briefly, 0.50 mL of methanolic extract of sample was diluted with 1.50 mL of distilled water and 0.50 mL of 10% (w/v) aluminium chloride added along with 0.10 mL of 1 M potassium acetate and 2.80 mL of distilled water. This mixture was incubated at room temperature for 30 min. The absorbance of resulting reaction mixture was measured at 415 nm UV-VIS spectrophotometer (Hitachi U-2001). Quantification of flavonoids was done on the basis of standard curve of quercetin prepared in 80% methanol and results were expressed in milligram quercetin equivalent (QE) per gram fw of fruits.

### 2.6. Antioxidant Activity

#### 2.6.1. Radical Scavenging Activity (ABTS Assay)

Total antioxidant activity was measured by improved ABTS method described by Cai et al. [32]. ABTS salt (7.0 *μ*M) and potassium persulfate (2.45 *μ*M) was added for the production of ABTS cation (ABTS^·+^) and kept in dark for 16 h at 23°C. ABTS^·+^ solution was diluted with 80% (v/v) ethanol till an absorbance of 0.700 ± 0.005 at 734 nm was obtained. Diluted ABTS^·+^ solution (3.90 mL) was added in 0.10 mL of methanolic extract and the resulting mixture was mixed thoroughly. Reaction mixture was allowed to stand for 6 min in dark at 23°C and absorbance was recorded at 734 nm using UV-VIS spectrophotometer. Samples were diluted with 80% (v/v) methanol to obtain 20–80% reduction in absorbance at 734 nm with respect to blank that was prepared with 0.10 mL 80% (v/v) methanol. A standard curve of various concentrations of ascorbic acid was prepared in 80% v/v methanol for the equivalent quantification of antioxidant potential with respect to ascorbic acid. Results were expressed in millimole (mM) ascorbic acid equivalent (AAE) per 100 g fw of fruits.

#### 2.6.2. Radical Scavenging Activity (DPPH Assay)

Traditional DPPH assay as described by Brand-William et al. [33] was modified for this study. An amount of 25 mL of 400 mM DPPH was added in 25 mL of 0.2 M MES buffer (pH 6.0 adjusted with NaOH) and 25 mL 20% (v/v) ethanol. DPPH cation solution (2.7 mL) was mixed with 0.9 mL sample extract and kept in dark at room temperature for 20 min. Reduction in the absorbance at 520 nm was recorded by UV-VIS spectrophotometer. Results were expressed in millimole (mM) ascorbic acid equivalent (AAE) per 100 g fw of fruits.

#### 2.6.3. Reducing Power (FRAP) Assay

Ferric reducing antioxidant power (FRAP) assay was performed following Benzie and Strain [34] with some modifications. FRAP reagent was prepared by adding 10 vol. of 300 mM acetate buffer (i.e., 3.1 g of sodium acetate and 16 mL glacial acetic acid per liter), 1 vol. of 10 mM 2,4,6-tri-2-pyridyl-1,3,5-triazin (TPTZ) in 40 mM HCl and 1 vol. of 20 mM ferric chloride. The mixture was pre-warmed at 37°C and 3.0 mL of the mixture was added to 0.10 mL methanolic extract and kept at 37°C for 8 min. Absorbance was taken at 593 nm by UV-VIS spectrophotometer. A blank was prepared by ascorbic acid and results were expressed in millimole (mM) of ascorbic acid equivalent (AAE) per 100 g fw of fruits.

### 2.7. HPLC Analysis of Phenolic Compounds

One hundred and twenty microliters extract of each population was used in triplicate in high performance liquid chromatography (HPLC) system equipped with L-7100 series pump (Merck-Hitachi, Japan) and L-7400 series UV-VIS detector (Merck-Hitachi, Japan). Phenolic compounds were separated by using 4.6 × 250 mm i.d., 5 *μ*m, Purosphere; C8 column. The mobile phase used for the study was water, methanol and acetic acid in the ratio of 80 : 20 : 1 and flow rate was 0.8 mL/min in isocratic mode. The spectra of compounds (total seven) were recorded at 254 nm for gallic acid, catechin, ellagic acid and 3-hydroxybenzoic acid, 370 nm for caffeic acid and chlorogenic acid and 280 nm for *ρ*-coumaric acid. The identification of phenolic compounds was done with respect of the retention time of corresponding external standard. UV-VIS spectra of pure standard at different concentrations were used for plotting standard calibration curve. The repeatability of quantitative analysis was 3.5%. The mean value of content was calculated with ±SD. The result was expressed as milligram per 100 g fw of fruits.

### 2.8. Statistical Analysis

All determinations of total phenols, flavonoids, antioxidant capacity by ABTS, DPPH, FRAP assay were conducted in five replicates. Phenolic compounds were measured in triplicates. The value for each sample was calculated as the mean ± SD. Analysis of variance and significant difference among means were tested by two way ANOVA using SPSS and Fisher's least significance difference (F-LSD) on mean values [35]. Correlation coefficients (*r*) and coefficients of determination (*r* ^2^) were calculated using Microsoft Excel 2007.

## 3. Results

### 3.1. Total Phenolic and Flavonoid Content

**Figure 1:**
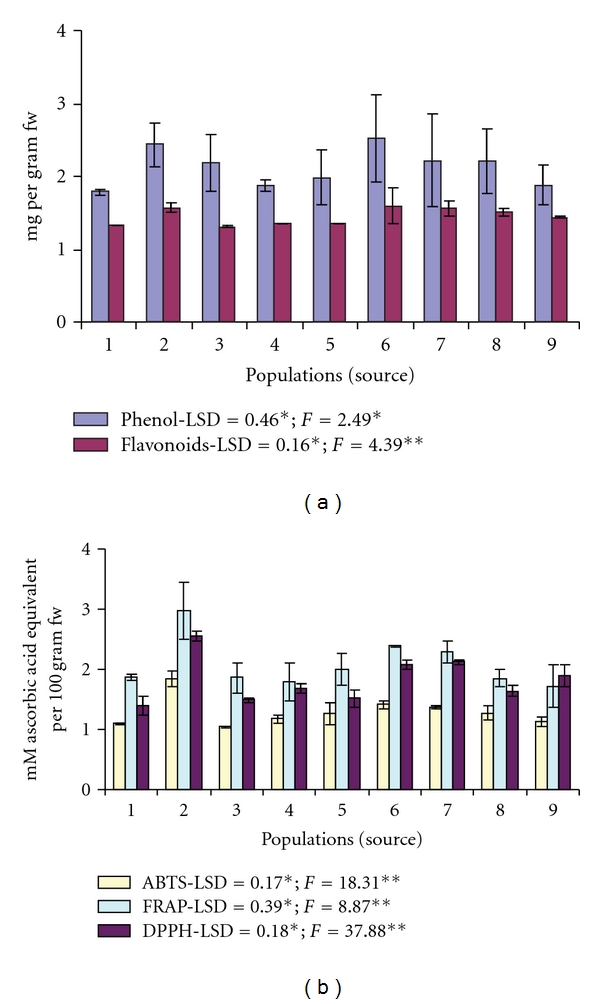
Total phenolic and flavonoids content (a) and antioxidant activity (b) of *M. esculenta* fruits; 1—Kalika; 2—Ayarpani; 3—Panuwanaula; 4—Jalna; 5—Dholichina; 6—Khirshu; 7—Shyamkhet; 8—Gwaldom; 9—Doonagiri; all values are mean of five measurements; LSD: least significance difference; levels of significance: *P < .05; **P < .01.

Total phenolic content in fruit extracts of *M. esculenta* varied between 1.78 mg GAE/gram (Kalika) and 2.51 mg GAE/gram fw (Khirshu) with an average value of 2.12 mg GAE/gram fw. ANOVA revealed significant variation in total phenolic contents (*F* = 2.49; *P* <  .05) across populations (Figure 1(a)). Total flavonoid contents ranged from 1.31 mg (Panuwanaula) to 1.59 mg (Khirshu) QE/gram fw, and variation across populations were significant (*F* = 4.39; *P* <  .01).

### 3.2. Antioxidant Activity

Antioxidant activity measured by three *in vitro* antioxidant assays, that is, free radical-scavenging ability by using ABTS radical cation (ABTS assay), DPPH radical cation (DPPH assay) and FRAP assay showed significant (*P* <  .01) variation among populations (Figure 1(b)). As compared to other populations, fruits obtained from Ayarpani population exhibited significantly more (*P* <  .05) antioxidant activity in all the three antioxidant assays (ABTS—1.84 mM; DPPH—2.55 mM; FRAP—2.97 mM AAE/100 g fw).

### 3.3. HPLC Analysis of Phenolic Compounds

**Figure 2:**
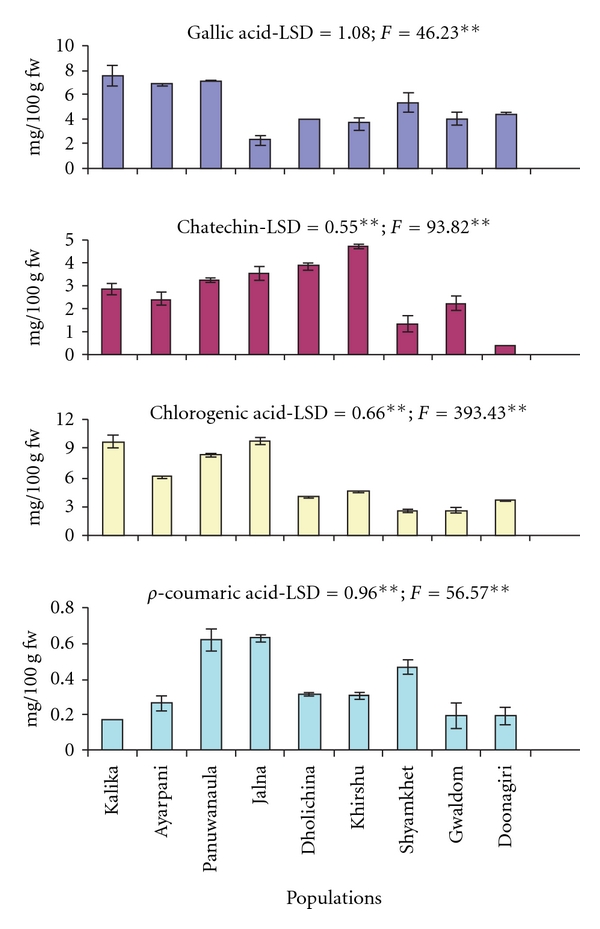
Phenolic compounds quantified by HPLC in *M. esculenta* fruits; all values are mean of five measurements; LSD—least significance difference; levels of significance: **P < .01.

Of the seven phenolic compounds used for HPLC analysis, only four (i.e., gallic acid, catechin, chlorogenic acid and *ρ*-coumaric acid) were detected in fruit extract of *M. esculenta.* These compounds showed significant (*P* <  .01) variation across the populations (Figure 2). Quantity of chlorogenic acid was highest (5.68 mg/g fw) followed by gallic acids (5.03 mg/100 g fw), catechin (2.72 mg/100 g fw) and *ρ*-coumaric acid (0.35 mg/100 g fw). While considering the fruits of different origin (i.e., population), it was revealing that the quantity of detected phenolic compounds varied considerably and the difference between minimum and maximum values were about three times for gallic acid, thirteen times for catechin, four times for chlorogenic acid and *ρ*-coumaric acid. HPLC analysis detected only a small proportion (0.065%) of phenolics. While combining all the phenolic compounds, Kalika population showed highest total phenolics (20.23 mg/100 g, 0.14% of total phenolics). The lowest value was found for fruits of Doonagiri population (8.62 mg/100 g fw; 0.046% of total phenolics).

### 3.4. Relationship among Altitude, Antioxidant Assays, Total Phenolics, Flavonoids, and Phenolic Compounds

**Figure 3:**
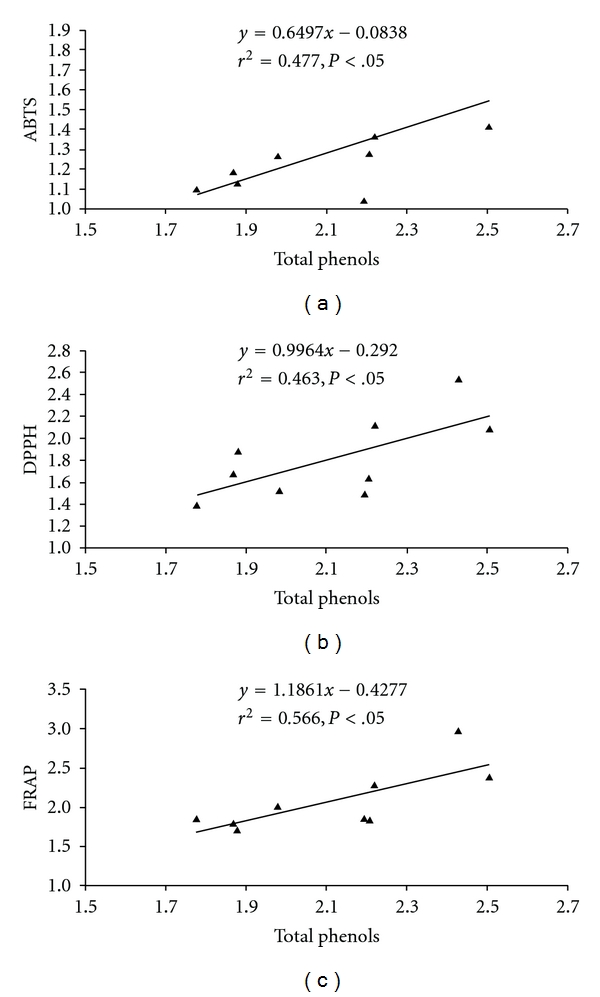
Relationship between total phenols and antioxidant activity of *M. esculenta* fruits following different *in vitro* assays (a) ABTS, (b) DPPH and (c) FRAP, (*n* = 9).

**Figure 4:**
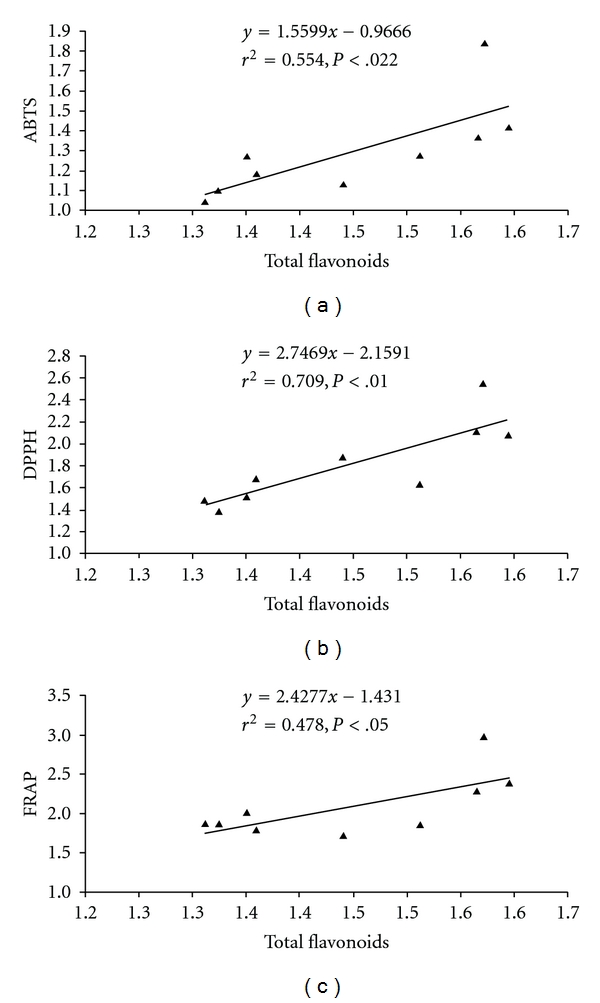
Relationship between total flavonoids and antioxidant activity of *M. esculenta* fruits following different *in vitro* assays (a) ABTS, (b) DPPH and (c) FRAP, (*n* = 9).

Considering altitude as an important independent variable in mountain areas, significant negative correlation with catechin (*r* = –0.778; *P* <  .05) was revealing. None of other compounds exhibited significant relationship with the altitude (Table 1). However, correlation matrix showed significant (*P* <  .05) positive impact of total phenolic and flavonoid contents on antioxidant activity (Table 1). Linear regression analysis revealed that phenolic contents contribute 46.3–47.6% of radical scavenging property (*r* ^2^  = 0.463 for DPPH and *r* ^2^  = 0.476 for ABTS) and 56.6% of reducing property (*r* ^2^  = 0.566) (Figure 3). Likewise, flavonoids contribute 55.4–70.9% radical scavenging property (*r* ^2^  = 0.554 for ABTS and *r* ^2^  = 0.709 for DPPH) and 47.8% of reducing property (*r* ^2^  = 0.478) (Figure 4). Among antioxidant assays, a strong positive relationship (*P* <  .01) was observed. The results showed that all three *in vitro* antioxidant assay, used in this study, were comparable and exhibited suitability for the species. The compounds present in the methanolic extract of *M. esculenta* fruits were capable of scavenging ABTS^·+^ and DPPH^·^ radical and also to reduce the ferric ions.

## 4. Discussion

**Figure 5:**
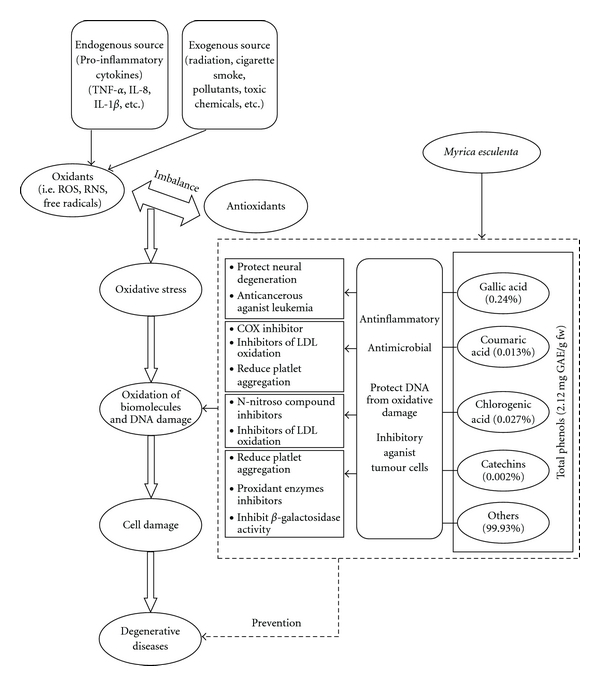
Hypothetical diagram explaining potential of *M. esculenta* for preventing oxidation of biomolecules, DNA damage and degenerative diseases.

Generally, it is believed that the reactive oxygen species (ROS), reactive nitrogen species (RNS) and free radicals in the body are generated through exogenous (radiation, cigarette smoke, atmospheric pollutants, toxic chemicals, over nutrition, changing food habits, etc.) and/or endogenous sources (pro-inflammatory cytokines— tumor necrosis factor-alpha (TNF-*α*), interleukin-8 (IL-8), interleukin-1B (IL-1B), etc. [36]). The free radicals, which are known to maintain homeostasis at the cellular level and work as signaling molecules, in excess are reported to result in oxidative stress [37] and cause various degenerative diseases [38]. In this context, antioxidants play an important role in prevention, interception and repairing of the body by stopping the formation of ROS, radical scavenging and repairing the enzymes involved in the process of cellular development [39]. Phenolics and flavonoids of plant origin are reported to have potent antioxidants and homeostatic balance between pro-oxidant and anti-oxidants is known to be important for maintenance of health as well as prevention from various degenerative diseases (Figure 5).

Considering the target species, the mean value for total phenolic content of *M. esculenta* fruits (2.12 mg GAE/gram fw) was comparables with values (0.94–2.82 mg/g) reported for fruit extract of different cultivars of *M. rubra* [25]. As compared with *M. rubra*, target species (*M. esculenta*) possess slightly more flavonoid contents. Therefore, presence of the phenolics and flavonoid contents in relatively higher amount in *M. esculenta* fruits would justify its comparative advantage over *M. rubra*. As such, phenolics and flavanoids constitute major group of compounds which act as primary antioxidants [40] and are known to react with hydroxyl radicals [41], superoxide anion radicals [42], lipid peroxyradicals [43], protect DNA from oxidative damage, inhibitory against tumor cell and possess anti-inflammatory and antimicrobial properties. The variations in phenolic and flavonoid contents across populations may be attributed to morphological as well as biochemical characters of the fruits. This would, however, suggest source specific variation of antioxidant potential.

All of the detected phenolic compounds, albeit detected in very small proportions (0.065%), are known to have antioxidant properties. Gallic acid, which is efficiently absorbed in human body, shows positive effect against cancer cell under *in vitro* condition [44]. Chlorogenic acid, a very common phenolic acid present in fruits [45], and catechin are effective in preventing oxidative injuries in human epithelial cells under *in vitro* [46]. As such, catechins form an important group of compound in the Mediterranean diet [47]. *ρ*-coumaric acid is believed to reduce the risk of stomach cancer by reducing the formation of carcinogenic nitrosamines [48]. Specific function of each detected compound in *M. esculenta* fruits is summarized (Figure 5), thereby, highlighting antioxidant potential of the species.

Significant scavenging and reducing capacity of the fruits extract was revealing in different methods. Similar studies on *M. rubra* fruit extracts have established variation in antioxidant activity (1.39–6.52 mM Trolox equivalent antioxidant capacity) across cultivars [25]. However, higher antioxidant capacity has been reported in *M. rubra* fruit extract using DPPH and FRAP assays [27]. While considering relationship of phenolic content and antioxidant activity, the established scavenging (45–70%) and reducing (48–55%) capacity of *M. esculenta* fruits are indicative of their strength as an antioxidant. The remaining antioxidant activity may be attributed to other phytochemicals like anthocyanins, vitamins, carotenoids, and so forth. The reports on *M. rubra* have established that cyanidin-3-*o*-glucoside, a major anthocyanin present in the species, was responsible for 12–82% of total antioxidant activity [27].

Strong positive relationship (*P* <  .01) of antioxidant assays suggested that all three *in vitro* antioxidant assays used in this study are comparable and exhibit their suitability for the species. The compounds present in the methanolic extract of the fruits of *M. esculenta* are not only capable for scavenging of ABTS^·+^ and DPPH^·^ radical but also to reduce the ferric ions. Similar strong positive correlation of DPPH free radical scavenging ability and ferric ion reducing ability are known in wines [49] and *Ilex kudingcha* [50]. These results support the basic concept that antioxidants are reducing agents.

## 5. Conclusion

Conclusively, results of this study signify that the extract of *M. esculenta* fruit is an important source of natural antioxidants which can play vital role in reducing the oxidative stress and preventing from certain degenerative diseases. Purification of the extract may lead to increased activity of the compounds. On a broader perspective, considering the remoteness and poor rural settings of Uttarakhand Himalaya in India, consumption of *M. esculenta* fruits is likely to benefit by scavenging and reducing free radicals in the body of rural inhabitants. However, observations of significant variations in antioxidant potential and phenolics across populations can be utilized gainfully for identification of best provenances for promotion under large scale plantation through horticulture and forestry interventions.

## Funding

GBPIHED in-house project (project no. 10).

## Figures and Tables

**Table 1:** Correlation matrix between altitude, total phenols, total flavonoids and antioxidant activity measured by different assays in selected populations of *M. esculenta* (*n* = 9).

*r*-value^a^	Altitude	Total phenols	Flavonoids	ABTS	DPPH	FRAP
Altitude	1					
Total phenols	−0.360	1				
Flavonoids	0.004	0.771*	1			
ABTS	0.057	0.691*	0.744*	1		
DPPH	0.176	0.68*	0.843**	0.878**	1	
FRAP	−0.132	0.753*	0.691*	0.949**	0.856**	1
Gallic acid	−0.165	0.057	0.078	0.017	0.264	0.078
Catechin	−0.778*	0.256	0.036	−0.215	0.130	0.036
Chlorogenic acid	−0.379	−0.404	−0.293	−0.371	−0.188	−0.293
*ρ*-Coumaric acid	−0.101	0.019	0.078	0.017	0.264	0.078

^a^Correlation coefficient.

Levels of significance: *P < .05; **P < .01.
